# Developing Methods for Assessing Mental Activity Using Human-Smartphone Interactions: Comparative Analysis of Activity Levels and Phase Patterns in General Mental Activities, Working Mental Activities, and Physical Activities

**DOI:** 10.2196/56144

**Published:** 2024-06-17

**Authors:** Hung-Hsun Chen, Chen Lin, Hsiang-Chih Chang, Jen-Ho Chang, Hai-Hua Chuang, Yu-Hsuan Lin

**Affiliations:** 1 Department of Mathematics Fu Jen Catholic University Taipei Taiwan; 2 Program of Artificial intelligence and Information Security Fu Jen Catholic University Taipei Taiwan; 3 Department of Biomedical Sciences and Engineering National Central University Taoyuan City Taiwan; 4 Institute of Population Health Sciences National Health Research Institutes Miaoli County Taiwan; 5 Institute of Ethnology Academia Sinica Taipei Taiwan; 6 Department of Psychology National Taiwan University Taipei Taiwan; 7 College of Medicine Chang Gung University Taoyuan Taiwan; 8 Department of Family Medicine Chang Gung Memorial Hospital Taoyuan Taiwan; 9 Department of Industrial Engineering and Management National Taipei University of Technology Taipei Taiwan; 10 School of Medicine National Tsing Hua University Hsinchu Taiwan; 11 Department of Psychiatry National Taiwan University Hospital Taipei Taiwan; 12 Department of Psychiatry, College of Medicine National Taiwan University Taipei Taiwan

**Keywords:** digital phenotyping, human-smartphone interaction, labor or leisure, machine learning, mental activity, physical activity

## Abstract

**Background:**

Human biological rhythms are commonly assessed through physical activity (PA) measurement, but mental activity may offer a more substantial reflection of human biological rhythms.

**Objective:**

This study proposes a novel approach based on human-smartphone interaction to compute mental activity, encompassing general mental activity (GMA) and working mental activity (WMA).

**Methods:**

A total of 24 health care professionals participated, wearing wrist actigraphy devices and using the “Staff Hours” app for more than 457 person-days, including 332 workdays and 125 nonworkdays. PA was measured using actigraphy, while GMA and WMA were assessed based on patterns of smartphone interactions. To model WMA, machine learning techniques such as extreme gradient boosting and convolutional neural networks were applied, using human-smartphone interaction patterns and GPS-defined work hours. The data were organized by date and divided into person-days, with an 80:20 split for training and testing data sets to minimize overfitting and maximize model robustness. The study also adopted the M10 metric to quantify daily activity levels by calculating the average acceleration during the 10-hour period of highest activity each day, which facilitated the assessment of the interrelations between PA, GMA, and WMA and sleep indicators. Phase differences, such as those between PA and GMA, were defined using a second-order Butterworth filter and Hilbert transform to extract and calculate circadian rhythms and instantaneous phases. This calculation involved subtracting the phase of the reference signal from that of the target signal and averaging these differences to provide a stable and clear measure of the phase relationship between the signals. Additionally, multilevel modeling explored associations between sleep indicators (total sleep time, midpoint of sleep) and next-day activity levels, accounting for the data’s nested structure.

**Results:**

Significant differences in activity levels were noted between workdays and nonworkdays, with WMA occurring approximately 1.08 hours earlier than PA during workdays (*P*<.001). Conversely, GMA was observed to commence about 1.22 hours later than PA (*P*<.001). Furthermore, a significant negative correlation was identified between the activity level of WMA and the previous night’s midpoint of sleep (*β*=–0.263, *P*<.001), indicating that later bedtimes and wake times were linked to reduced activity levels in WMA the following day. However, there was no significant correlation between WMA’s activity levels and total sleep time. Similarly, no significant correlations were found between the activity levels of PA and GMA and sleep indicators from the previous night.

**Conclusions:**

This study significantly advances the understanding of human biological rhythms by developing and highlighting GMA and WMA as key indicators, derived from human-smartphone interactions. These findings offer novel insights into how mental activities, alongside PA, are intricately linked to sleep patterns, emphasizing the potential of GMA and WMA in behavioral and health studies.

## Introduction

Human biological rhythms are commonly assessed through the measurement of physical activity (PA), such as wrist-worn actigraphy, which estimates sleep and wake times based on patterns of PA [1]. Actigraphy uses wrist-worn accelerometers to collect data on individuals’ physical activities, providing a practical means to approximate sleep and circadian rhythm measurements. The rest-activity rhythm throughout the day serves as a valuable metric for quantifying circadian rhythms [2]. However, human beings distinguish themselves from other animals by engaging in highly active mental activity, particularly those individuals who endure long working hours in contemporary society. Given its greater quantity and relevance, mental activity may offer a more substantial reflection of human biological rhythms than PA alone. This is especially pertinent in the digital age, where mental engagement often supersedes PA in daily life. Further differentiation within mental activity can be observed through various theories related to labor or leisure and cognitive control [3,4]. Nevertheless, the current landscape lacks continuous methods for measuring mental activity and working-related mental activity throughout the day. Although some studies have endeavored to explore these aspects, their ecological validity often faces challenges stemming from prolonged monitoring, reliance on self-reports, and limited accuracy [5].

In response to these challenges, our study addressed the nuanced relationship between mental activities and daily interactions with technology, especially with smartphones. We advanced a novel methodology centered on human-smartphone interaction to quantify both general mental activity (GMA) and working mental activity (WMA). This method notably distinguished between work and leisure periods based on interaction patterns. By doing so, our study bridges a significant gap in understanding how modern technology influences daily mental rhythms, a topic underexplored in current literature.

Previous research in cognitive psychology [3,4,6-9], which focused on the dynamics of mental effort between labor and leisure, primarily used experience-sampling studies. However, these approaches often fell short of capturing the complex interplay of smartphone use across different stages of mental engagement. Additionally, the dual role of smartphones as tools for work and leisure blurred the distinction between professional and personal use, a phenomenon that prior studies had noted [10]. For example, smartphones were frequently used for work-related purposes at home and for private purposes at work, reflecting their role as leisure surrogates. Nevertheless, the true value of a smartphone in these contexts was less about the device itself and more about the behaviors it facilitated, such as responding to notifications [8]. Traditional experience-sampling methods struggled to categorize and analyze these screen events in terms of their relevance to work-related or GMAs.

To overcome these challenges, our study used a more nuanced approach, building on our previous work where we developed an application named Staff Hours [6]. This app harnessed GPS background data to automatically calculate users’ work hours while also continuously recording screen events, including time stamps of notifications, screen on/off instances, and app use. This rich data collection led us to embrace the concept of “digital phenotyping”—the moment-by-moment quantification of the individual-level human phenotype in situ, using data from personal digital devices [5,9,11,12]. Using this innovative approach, our previous studies successfully identified various behaviors linked to digital interactions, such as sleep-wake cycles [13], circadian rhythms [14], and patterns of problematic smartphone use [15]. This methodology allowed for a more nuanced analysis of smartphone interactions, transcending the limitations of conventional experience-sampling studies. By continuously recording screen events, we were able to more accurately assess the complex relationship between smartphone interactions and the user’s mental state, whether related to work or leisure.

In this study, we used standard wrist-worn actigraphy to assess sleep-wake cycles while simultaneously recording the time stamps of human-smartphone interaction patterns through our developed app. This app automatically captures the screen events associated with human-smartphone interaction. We devised a method that uses an algorithm similar to actigraphy to calculate the biological rhythms derived from human-smartphone interaction, which we interpret as “general mental activity.” Additionally, we developed a machine-learning algorithm that combines human-smartphone interaction patterns with GPS-defined work hours to generate probabilities indicating engagement in work-related tasks, which we interpret as “working mental activity.” Our study aims to compare these 2 novel methods, GMA and WMA, developed by our team, with standard actigraphy serving as the reference for measuring PA. Both the GMA and WMA methods passively and continuously capture data, exhibiting similar characteristics to PA measurements. We would investigate the hypotheses that (1) the phase of the 3 activities would differ, (2) activity levels might vary between workdays and nonworkdays, and (3) we would identify mutual influences between activity levels, such as the impact of sleep on WMA and the influence of sleep duration on PA levels.

## Methods

### Participants

A total of 24 health care professionals (21 females; mean age 39.7, SD 8.3 years) were recruited from a workplace health promotion program for medical staff at 7 hospitals in northern Taiwan, for the study period between September 2021 and February 2023. Of the 24 participants in this study, 14 participants were health care professionals involved in primary care, including physicians, nurses, and dietitians. The remaining 10 participants were administrative staff not directly involved in patient care. All participants worked in hospital environments, with none engaged in remote work. Their job nature was classified as office work rather than physical work, and none of the participants were engaged in shift work during the study period. Prior to participation, all individuals provided informed consent. Eligible participants were required to have an Android operating smartphone that would be exclusively used by them throughout the study period.

**Figure 1:**
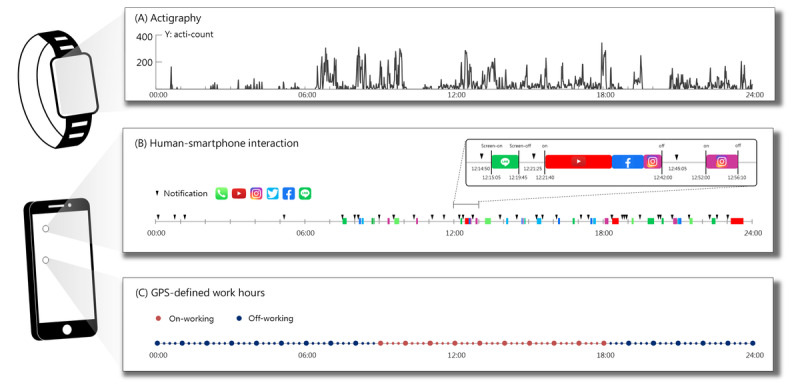
The measurement methods in the study. (A) Physical activity was recorded using a wrist-worn actigraphy device. (B) Records of human-smartphone interaction were captured using the Staff Hours app. The recorded events included timestamps indicating the type of app used, screen on/off, and notifications. (C) The Staff Hours app also recorded the GPS location data, enabling the identification of work hours based on the user’s location at their workplace and nonworkplace locations.

Participants were instructed to install the Staff Hours app on their smartphones and wear a wrist actigraphy device continuously for a minimum of 1 week (Figure 1). Throughout the study, which involved 24 participants, a collective duration of 481 person-days was recorded. It is worth noting that there were 24 days during this period where no smartphone use data were recorded at all. Therefore, the analysis was conducted on a data set of 457 person-days, comprising 323 workdays and 115 nonworkdays.

The study used only Android users due to technical limitations. The use of only Android smartphones was due to technical limitations on iOS devices, which at the time only allowed the collection of GPS data. Unlike Android, the iOS version did not support the retrieval of detailed human-smartphone interaction patterns such as time stamps of screen on/off, notifications, and app use types. These data points were critical for calculating GMA and WMA in our study. It was ensured that participants did not share their smartphones with other individuals during the study period. While wearing the actigraphy device was mandatory, no specific instructions were given regarding the placement of smartphones.

### Ethical Considerations

The study adhered to the principles set forth in the Declaration of Helsinki. The institutional review boards of Chang-Gung Memorial Hospital (202100434B0A3 and 202002452A3) approved the research protocol. All participants provided informed consent prior to their inclusion in the study. They signed consent forms that specifically addressed the use and publication of their data. Regarding privacy and confidentiality, all data were deidentified to ensure the protection of participant information. No additional compensation was provided to the participants for their involvement in this study. This decision was made clear to the participants during the informed consent process, ensuring transparency and fairness in the study’s conduct.

### Study Design

#### Physical Activity

Participants were instructed to wear a research-grade wrist actigraphy device (MiCorTM A100, MiTAC Inc, Taiwan) on their nondominant wrist. This device collected data on acceleration across 3 axes, with an internal sampling rate of 30 Hz. The actigraphy watches recorded accelerations along 3 axes, which were combined using the Euclidean distance of the accelerations’ deviation from zero (Z). The data were then bandpass filtered within the range of 0.5-3 Hz. The Z values exceeding a predefined threshold were integrated over 2 seconds, and 1-second epochs of activity counts (acti-counts) were derived by averaging the integrated segments within 1 minute [1,16].

To estimate sleep and wake times, we used the standard Cole-Kripke algorithm on the acti-count data with slight modifications [16]. A crucial component of this algorithm, the minute-by-minute categorization of data into rest-active states, based on the weighted sum of the current minute with that of the contiguous minutes, was extracted to investigate the PA state during smartphone use. MATLAB (MathWorks) was used for the implementation of the algorithm using preexisting codes.

#### General Mental Activity

The Staff Hours app automatically recorded various smartphone events: GPS location data, time stamps of screen on/off events, notifications, and the labels of apps used in the background, all while maintaining a power-saving design. Using geofencing technology, it automatically logged the work hours spent in the workplace. The accuracy of the app-recorded work hours, with a sensitivity of 94.6% and specificity of 93.9%, was validated in our previous study [6]. Users were able to view their work hours on the app and, for enhanced accuracy and flexibility, adjust their actual work hours within a 7-day window.

These recordings formed time series of screen events, including notifications, screen on/off events, and app use. These data effectively charted human-smartphone interactions with significant temporal stability, as highlighted in [17]. Moreover, these time stamps reflected stimuli, individual responses, and content types typically examined in laboratory settings [5]. By harnessing the advanced data recording capabilities of smartphones, we successfully measured these interactions continuously and precisely in ecologically valid, real-world environments.

**Figure 2:**
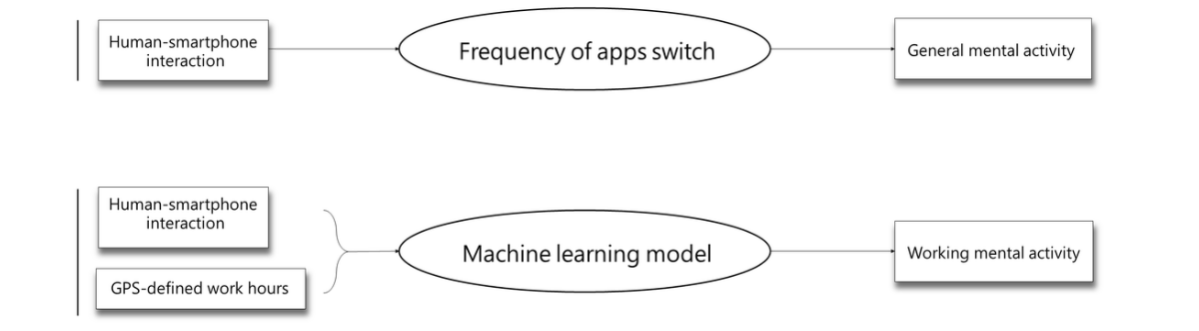
Methods for calculating general mental activity (GMA) and working mental activity (WMA). Human-smartphone interaction records were analyzed to determine the frequency of app switches, used as a proxy for GMA. These data were then used to simulate activity levels comparable to those obtained from the wrist actigraphy device. WMA was assessed by calculating the probability of an individual being in work mode using human-smartphone interaction patterns and corresponding GPS location data.

The use counts were defined as the number of apps used per minute (Figure 2). To account for the potentially patchy nature of app use behavior compared with activity data, we used longer durations to represent app use behaviors, referred to as “app-counts.” App-counts were obtained by summing up the minute-by-minute use counts in 5-minute nonoverlapping epochs (288 epochs per day) to avoid excessive segments with zero counts. These data were then used to mimic the activity data obtained from the wrist actigraphy device.

#### Working Mental Activity

To evaluate WMA, we developed a model that calculated the probability of an individual being in work mode, using human-smartphone interaction patterns and GPS location data.

**Figure 3:**
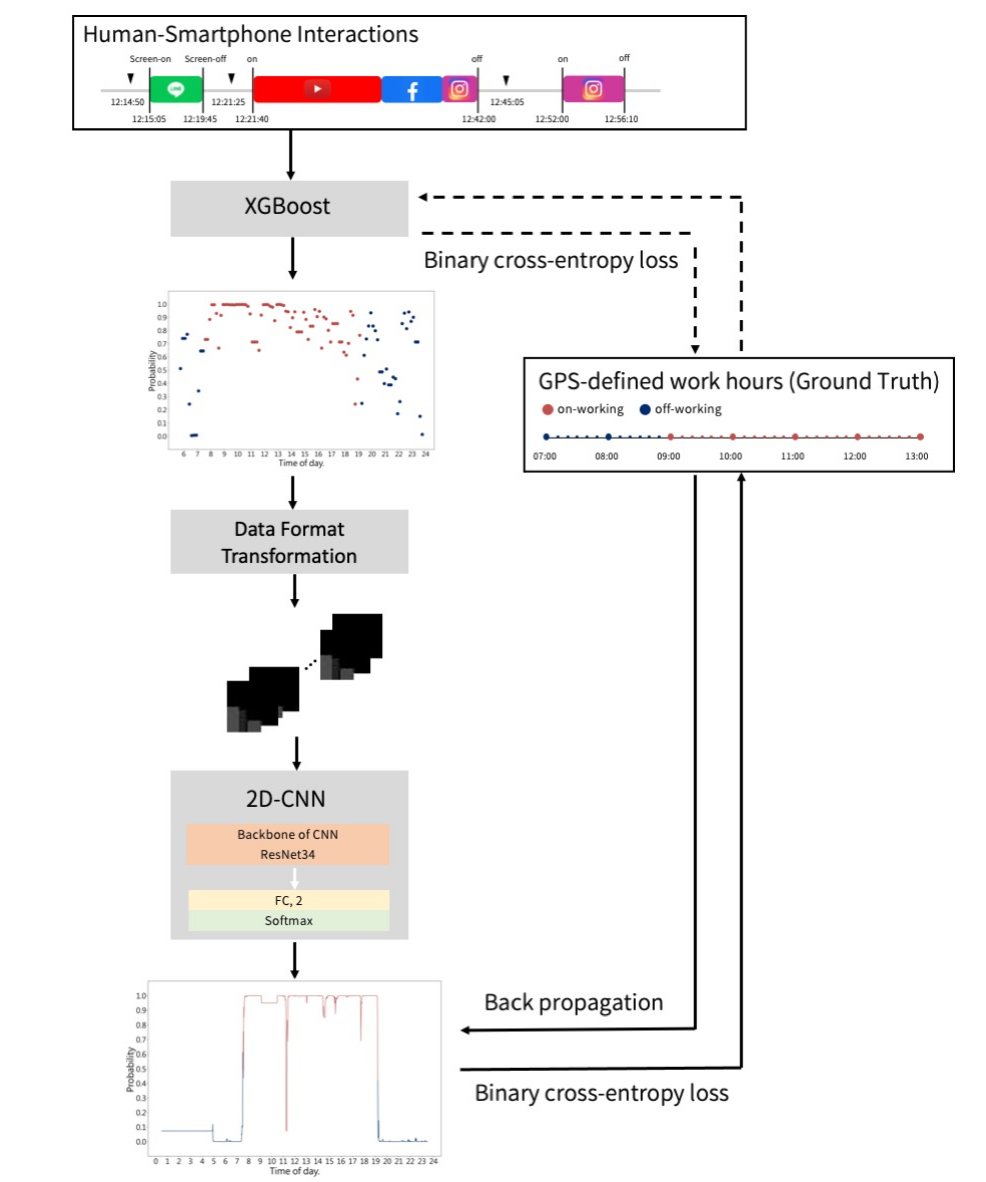
The algorithm to generate working mental activity. Smartphone use logs, that is, human-smartphone interaction patterns, correlated with GPS data, establish the context of working and nonworking periods. An advanced machine learning model, integrating XGBoost and a 2D convolutional neural network (2D-CNN) processes these inputs. (Further details are also referred to in Multimedia Appendix 1).

The process was divided into 2 stages. In the first stage, extreme gradient–boosted trees were applied to transform interaction patterns into probabilities [18]. Subsequently, given a threshold, we calculated the length of consecutive time during which the probability values differed by less than the threshold before and after each time point. Ultimately, the transformed values were used as the grayscale values for that time. Data were then converted into 30×30–pixel images using 30-minute intervals as the unit of time. In the second stage, a 2D convolutional neural network model used these 30×30 grayscale images to train a binary classification model (distinguishing between “at work” and “off work”). The neural network architecture was based on ResNet-34 for feature extraction and was fine-tuned accordingly. Ultimately, the model could generate a sequence of continuous probabilities based on the previous probabilities in the sequence, as illustrated in Figure 3 and Multimedia Appendix 1.

During the training phase for WMA, models were individually tailored for each participant. We excluded 5% (n=24) of person-days from our analysis due to the complete absence of smartphone use data on these days. For the training of individual user models, data were integrated by the minute, and a 10-minute time window was set to generate the training data set. Given the sequential nature of user data, we sorted it by date and divided it into person-days in an 80:20 ratio (80% for the training data set and 20% for the testing data set). As a result, the average size of the testing data set per user was 6.4 days, with the smallest data set comprising 2 days. The accuracy of the testing data set was minutely assessed, ensuring at least 2760 data points per user to calculate accuracy. We applied the same algorithm across data sets for 24 participants.

In the testing data set, the model demonstrated the following performance metrics: the average accuracy was 0.89 (SD 0.08), the average precision was 0.84, the average recall was 0.79 (SD 0.21), and the average *F*_1_-score was 0.79 (SD 0.18). The average area under the receiver operating characteristic curve (AUC) per participant was 0.8, ranging from 0.62 to 0.97. AUC calculations were deliberately performed using the testing data set, excluding the training set, to prevent overfitting and demonstrate the model’s robustness.

Machine learning algorithms were implemented using the Python scikit-learn library (Python Software Foundation), using 5-fold cross-validation. Missing values were imputed by sampling with replacement from nonmissing values. All data processing and analysis were performed using Python (version 3.8.5; Python Software Foundation), NumPy (version 1.19.2), scikit-learn (version 0.23.2), and PyTorch (version 2.0.1).

### Activity Levels

**Figure 4:**
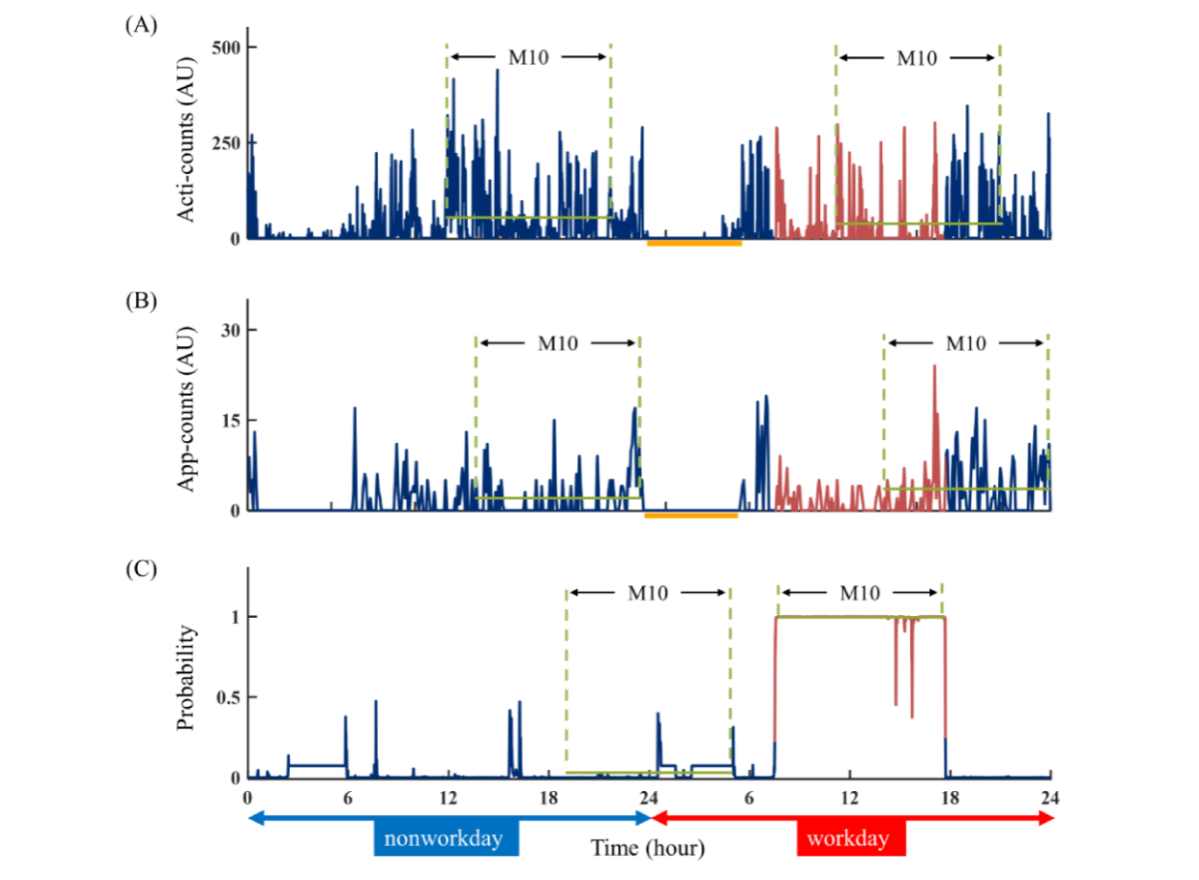
Comparison of physical activity (PA), general mental activity (GMA), and working mental activity (WMA). The figure presents the data from a single participant for 2 consecutive days, one being a nonworkday (represented in blue) and the other being a workday (represented in red). The color-coded segments indicate the time periods during which the participant was engaged in work or nonwork activities. To assess activity levels, we used the M10 value as a representative indicator, which encompasses (A) PA, (B) GMA, and (C) WMA, on a daily basis. The M10 value was derived by calculating the average acceleration during the 10-hour period (highlighted in green) with the highest activity level, providing insights into the participant’s peak activity during the day. Notably, there were significant differences observed in the M10 values of WMA between nonworkdays and workdays (0.030 vs 0.994). During workdays, the M10 period of WMA (7:39-17:39) occurred earliest, followed by PA (11:10-21:10), while GMA (13:32-23:32) exhibited the latest M10 period. Although the phase of PA is later than the phase of GMA, the calculated sleep periods (in orange) for both activities (23:43-05:28 vs 23:53-05:23) are remarkably similar.

In this study, we expanded the use of the M10 value as a representative indicator of the participant’s activity levels, encompassing PA, GMA, and WMA, on a daily basis (Figure 4). The M10 value was derived by calculating the average acceleration during the 10-hour period with the highest activity level, providing insight into the participant’s peak activity during the day. Through the application of the M10 value, we were able to consistently assess and compare the levels of PA, GMA, and WMA. This metric, widely acknowledged as an informative indicator of an individual’s PA level in previous studies [19,20], was successfully used in both the assessment of GMA and WMA within this study. The statistical distributions of the main variables in this study ranged from –0.53 to 0.96 for skewness, and –1.13 to 1.06 for kurtosis, all falling within the normal and acceptable range (absolute values<1.5) for further statistical analysis.

### Statistical Analysis

To examine the impact of employment status on activity levels, several statistical analyses were conducted. An independent *t* test compared PA, GMA, and WMA levels (M10 value) between workdays and nonworkdays, aiming to identify significant differences based on employment status. Additionally, a paired *t* test assessed agreement between actigraphy-measured sleep indicators and app-measured counterparts, detecting variations and evaluating consistency. Another independent *t* test compared sleep indicators specifically between workdays and nonworkdays, exploring differences associated with employment status.

**Figure 5:**
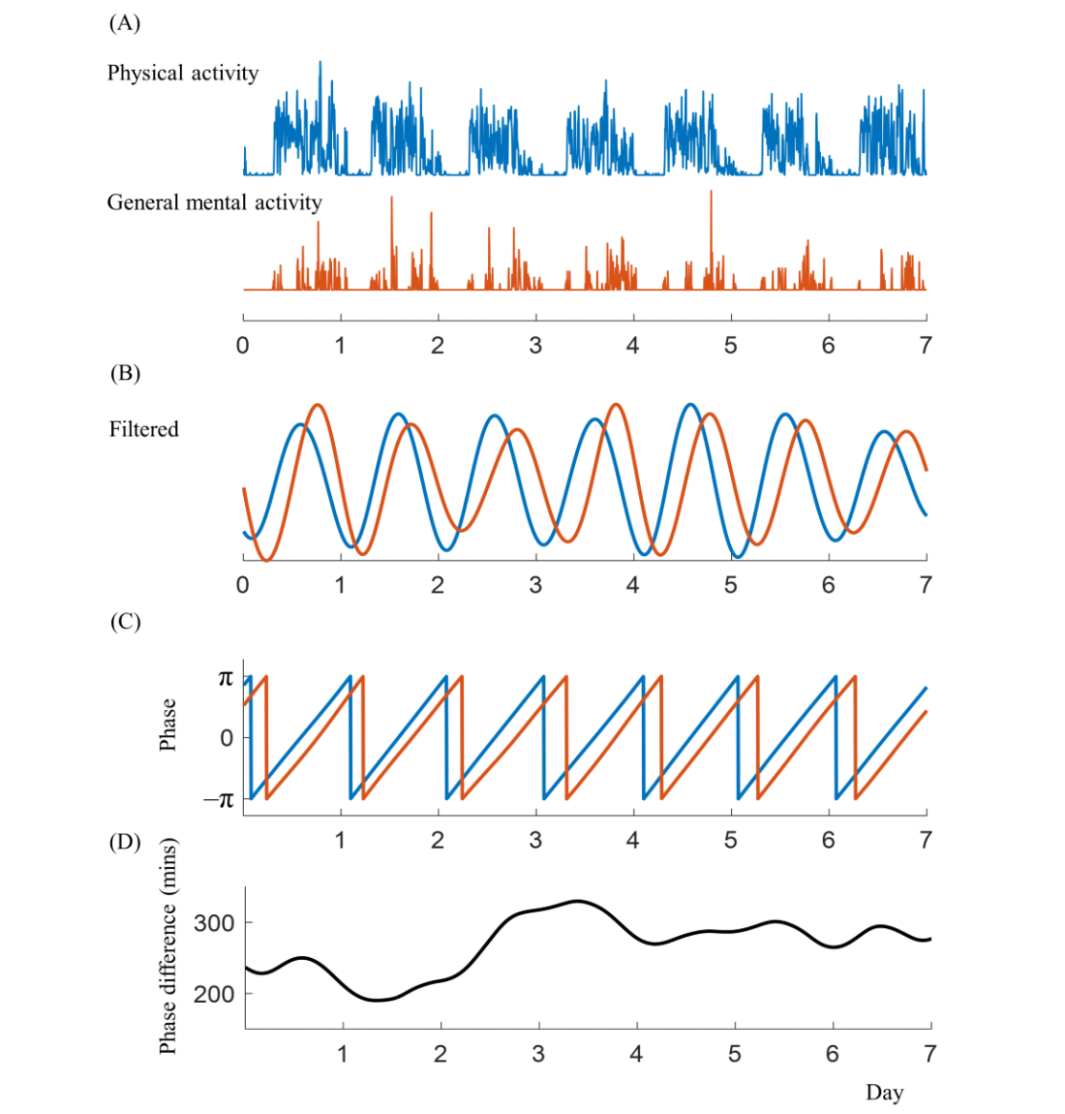
Phase difference calculation process. The illustrative example of calculating the circadian phase differences between physical activity and human-smartphone interaction patterns. (A) Raw data of physical activity (in blue) and general mental activity (in orange) generated by human-smartphone interaction patterns for a 7-day period of the same individual. (B) Filtered data for the cycles between 16 and 28 hours, retaining approximately 24-hour cycles of physical activity (in blue) and general mental activity (in orange). (C) Phase calculation using Hilbert transformation, showing the phase range of each cycle from –π to π. The graph shows that general mental activity (in orange) exhibits a later phase compared with physical activity (in blue). (D) Subtracting the phase of physical activity from the phase of general mental activity and converting the phase differences between physical activity and general mental activity to a 24-h time format (where 24 h=2π), the averaged phase difference reveals that general mental activity lags behind average physical activity by 268 min.

Phase difference quantifies the relative shift in phase between interacting signals r [21,22]. To calculate the phase difference (eg, PA and GMA), signals were filtered using a second-order Butterworth filter within a frequency range of 16-28 hours to extract circadian rhythms (Figure 5).

The filtered signal then undergoes a Hilbert transform, which can be represented as:



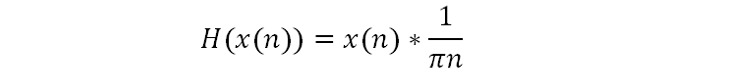



where * denotes the convolution operation. The Hilbert transform extracts the instantaneous phase of the filtered signal by getting the complex numbers of the resultant signals. Next, the phase of the reference signal is subtracted from the phase of the target signal to obtain the instantaneous phase difference:



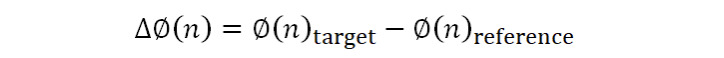



For example, when the reference is the GMA signal and the target signal is PA signal, we can evaluate whether the circadian of PA is advanced or delayed in associated with that of GMA. Finally, to obtain a representative measure, averaging is performed on the time-varying phase difference signal over a specific time interval:



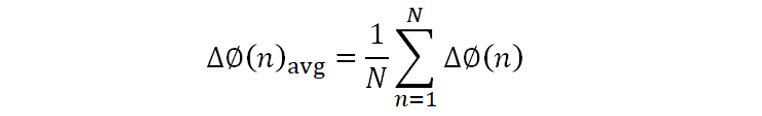



where *N* is the number of samples. This averaging step provides a more stable and meaningful measure of the overall phase relationship between the 2 signals, reducing the influence of short-term fluctuations or noise.

Considering the impact of sleep quality on daily performances, this study examined the effects of sleep indicators on mental activities. Using multilevel modeling via SPSS (version 25; IBM Corp) mixed model due to the nested data structure (daily information within each participant), we predicted the next day’s PA, GMA, and WMA levels (M10) based on the previous night’s sleep patterns (total sleep time and midpoint of sleep), controlling for participants’ demographic backgrounds (gender and age). In the multilevel modeling, the previous night’s sleep time, sleep midpoint, PA, GMA, and WMA levels are considered Level 1 data (within-person), while gender and age are considered Level 2 data (between-person).

## Results

There were significant differences in activity levels between workdays and nonworkdays for WMA, as indicated by the M10 value (0.774, SD 0.214 vs 0.153, SD 0.166; *P*<.001). However, no significant differences were observed for PA (73.5, SD 22.5 vs 72.3, SD 23.3; *P*=.60) and GMA (2.9, SD 1.9 vs 2.9, SD 2.0; *P*=.81) between workdays and nonworkdays.

The phase differences among the 3 activity levels were found to be significant. WMA exhibited an earlier phase than PA by 1.08 (SD 1.22) hours (*P*<.001), while PA showed an earlier phase than GMA by 1.22 (SD 1.22) hours (*P*<.001). However, when comparing sleep onset (23:41, SD 1:11 vs 23:35, SD 1:17; *P*=.14), wake time (6:45, SD 1:17 vs 6:43, SD 1:14; *P*=.73), and total sleep time (416.1, SD 77.8 min vs 416.8, SD 81.6 min; *P*=.89) using PA and GMA, there were no significant differences observed. Therefore, the sleep time estimated by PA was chosen for further analysis since actigraphy is the standard measurement for calculating long-term sleep-wake cycles [1].

The comparison between PA and GMA on workdays and nonworkdays revealed no significant phase differences. However, the sleep-wake cycle on nonworkdays tended to be delayed compared with workdays, with a later sleep onset by 23.0 (SD 7.7) minutes (*P*=.003) and a later wake time by 37.5 (SD 8.5) minutes (*P*<.001). Nevertheless, the total sleep time did not significantly differ between nonworkdays and workdays (412.7, SD 72.5 vs 425.5, SD 90.7; *P*=.13).

Table 1 displays the associations among different mental activities and sleep indicators from the previous night (total sleep time and midpoint of sleep) while controlling the participants’ demographics.

The results indicate a significant negative correlation between WMA’s activity level and the previous night’s midpoint of sleep. This suggests that a later bedtime and wake time were associated with lower activity levels in WMA on the following day. However, no significant correlation was found between WMA’s activity level and total sleep time. Additionally, no significant correlations were observed between the activity level (M10 value) of PA and GMA and the sleep indicators from the previous night.

**Table 1:** Multilevel model coefficients (β) of activity level (M10) and sleep indicators in the previous day.

Characteristics	Physical activity			General mental activity			Working mental activity	
	*β*	*P* value	*β*		*P* value	*β*		*P* value
Midpoint of Sleep	–0.079	.06	–0.026		.42	–0.263		<.001
Total sleep time	0.056	.19	–0.009		.78	–0.089		.07
Gender^a^	0.670	.11	–0.684		.15	–0.187		.42
Age	–0.017	.30	–0.034		.07	–0.007		.48

^a^Gender was dummy-coded as 1=male and 2=female.

## Discussion

### Principal Findings

In this study, we introduced a novel method based on human-smartphone interaction to assess GMA and WMA. Our approach offers a unique perspective on mental effort allocation, which has been a subject of scientific inquiry in cognitive psychology. Previous research has explored the labor or leisure tradeoff in cognitive control [3], often focusing on smartphone use at work as a proxy for leisure activity [8,10,23]. However, we argue that the definition of WMA is more explicit compared with the definition of leisure mental activity. Leisure tasks are typically described as unproductive and mentally undemanding relative to the cognitive demands of labor tasks [3]. Our study used human-smartphone interaction patterns, combined with workplace identification, to generate WMA measures. This approach provided a clear distinction between work-related and nonwork-related mental activity. By considering the specific context of WMA and incorporating smartphone interaction patterns within a defined workplace, our approach provides a more nuanced understanding of the allocation of mental effort.

Our study revealed a notable finding regarding the phase differences among WMA, PA, and GMA. Specifically, we observed that WMA occurred earlier in the day compared with PA, and PA occurred earlier than GMA. This finding correlates with the results reported by Dora et al [23], where participants were more likely to interact with their smartphones when fatigued or bored but did not necessarily use their smartphones for longer durations when experiencing higher levels of fatigue or boredom. The earlier occurrence of WMA compared with GMA can be explained by the possibility that participants, feeling fatigued from work, engaged in WMA activities before transitioning to GMA. This suggests that participants may have started using their smartphones after experiencing fatigue, corresponding to the active phase of WMA. However, once participants became actively engaged in GMA, characterized by smartphone use, there seemed to be a decrease in the activity levels of both PA and WMA. This finding is consistent with the results where participants reported increased fatigue and boredom after using their smartphones [23]. Consequently, following the active phase of GMA, the subsequent phase appears to be associated with sleep. These findings indicate a sequential pattern of mental and physical activities throughout the day.

In the method to describe WMA, we acknowledge the pervasive use of smartphones in modern-day work [7,10], particularly among health care professionals who heavily rely on these devices for their daily tasks. Hence, we used a machine learning model for WMA by associating human-smartphone interaction patterns with the context of the workplace. The introduction of smartphones has significantly transformed people’s lives, blurring the boundaries between work and private life [7,10]. While Dora et al [10] examined the smartphone use characteristics of employees, distinguishing work-related use at home from private use at work, our machine learning approach offers a more personalized distinction based on human-smartphone interaction patterns. By considering the context-incongruent purpose of smartphone use for each individual, our method provides a more nuanced understanding of the interplay between work and private life. Furthermore, our approach complements the findings in previous research [10] that the current value of the smartphone did not significantly contribute to predicting switches from labor to leisure when considering the current task value. However, participants reacted strongly to naturally incoming notifications, which strongly influenced labor-to-leisure switches. In our study, human-smartphone interaction patterns, including screen on/off, app type, and notifications, allowed us to observe participants’ smartphone use patterns and assess whether they reacted strongly to naturally incoming notifications, aligning with the findings in the previous study [10]. By using human-smartphone interaction patterns to generate WMA and considering the specific workplace context, our research methodology offers a more comprehensive understanding of the dynamics between work and private life in relation to smartphone use. These insights contribute to the ongoing discussion on the impact of smartphones on work-life boundaries and provide a basis for future investigations in this area.

Our study used an innovative method of assessing mental activity using human-smartphone interaction patterns. We directly computed GMA and WMA using this approach. Interestingly, we observed distinct patterns between GMA and WMA, with WMA exhibiting a significantly earlier phase compared with GMA, approximately 2 hours earlier. Furthermore, while GMA did not show differences between workdays and nonworkdays, WMA demonstrated significantly higher activity levels on workdays compared with nonworkdays. Although WMA was derived using a machine learning model, often referred to as a “black box,” which may be challenging to validate directly, the significant difference in activity levels between workdays and nonworkdays provides supporting evidence that WMA indicator in this study represents WMA. Additionally, WMA was not solely influenced by work-related time periods. Our findings indicate that WMA activity levels were influenced by delayed bedtime and wake time. Specifically, if individuals have a later bedtime and wake time, their average WMA activity levels during the 10 most active hours of the day will be lower. This finding aligns with the previous research suggesting a link between late sleep schedules, and decreased work efficiency the following day [24-27], and supports the notion that WMA captures the impact of sleep patterns on working-related mental activity.

### Limitations

Our study has several limitations that should be considered when interpreting the results. First, the small sample size and the use of convenience sampling focused exclusively on health care professionals may restrict the generalizability of our findings to other occupational groups. Additionally, the specific setting of the participants, who interacted with smartphones in a manner facilitated by GPS data for assessing WMA, might not accurately represent interaction patterns in a more diverse workforce where job roles and locations vary widely. Furthermore, the preliminary nature of these findings limits the scope and applicability of the research, suggesting that the conclusions drawn should be viewed as initial insights rather than definitive results. This emphasizes the need for further studies with broader and more varied samples to validate and expand upon these initial findings. Additionally, the WMA assessment’s reliance on the contrast between workplace and nonworkplace human-smartphone interactions might not directly translate to occupations without clear geographical boundaries or those that have experienced significant shifts to remote work arrangements. As we collected data during a period marked by the pandemic, from September 2021 to February 2023, our study inadvertently captured the implications of an extraordinary period where the health care professionals’ work patterns were more stable compared with other professions affected by the pandemic’s disruptions. It is, therefore, crucial to consider the extent to which the pandemic’s impact on work patterns across different industries may influence the applicability of our findings. As industries gradually return to prepandemic operational modes, the generalizability of our results may require further validation. These insights have been integrated into the discussion section of our paper to elucidate the specific occupational context of our participants during the pandemic and to underscore the need for the cautious application of our results to the general working population. Second, 5% (n=24) of the person-days could not be included in the analysis of GMA and WMA. This could be attributed to participants turning off their smartphones throughout the day, using different smartphones at work and off work, or relying on personal computers or tablets as alternatives to smartphones. Consequently, the probability in work mode failed to accurately differentiate between on-working and off-working situations in these cases. Third, the use of the Staff Hours app limited the study’s applicability to the Android operating system, and future research should consider compatibility with other operating systems. Fourth, we encountered challenges with potential interruptions in data collection from both the wrist-worn actigraphy device and the smartphone app, mainly due to low battery or nonuse by participants. To improve data robustness in future research, we plan to integrate the app with actigraphy. This approach aims to reduce data gaps and provide a more holistic perspective in our future studies. In addition, for activities where work locations are not fixed, our Staff Hours app allows for manual annotation, albeit this method is less consistent compared with the automatic 10-minute GPS logging due to potential human error or omissions. Fifth, the validation of the relationships between WMA, PA, and GMA was not fully achieved in this study. Despite observing no statistically significant differences in sleep onset and wake times measured by PA and GMA, GMA, when assessed against the widely accepted standard of actigraphy validated for PA, was confirmed to be reliable in assessing sleep dimensions. This result was also consistent with our previous research findings [28]. Furthermore, the performance of WMA in this study on the testing data set validated the use of human-smartphone interaction patterns to distinguish between work and nonwork locations, as the average AUC per participant was 0.80, ranging from 0.62 to 0.97. However, further research is needed to validate the relationships between WMA, PA, and GMA through interviews or additional qualitative methods with the participants. Finally, all participants were blinded to the locally stored records to minimize “biofeedback” effects, which could lead to self-recognition and behavioral modification. This blinding was crucial to ensure the authenticity of the data collected, as awareness of being monitored might have influenced the participants’ natural behavior patterns. However, this approach also limited our ability to gather immediate, subjective feedback from participants about their experiences and the results obtained. While this might be seen as a limitation, it was a necessary tradeoff to preserve the integrity and ecological validity of the data. Future studies could explore the feasibility of incorporating participant feedback mechanisms that do not compromise the naturalistic observation essential to our research objectives. Despite these limitations, our study serves as a stepping stone toward comprehensive investigations into the neurobehavioral aspects of human-smartphone interaction.

### Conclusions

In conclusion, our study introduced a novel approach to assessing mental activity using human-smartphone interaction patterns. The observed phase differences among WA, PA, and GMA shed light on the temporal organization of activities throughout the day.

## Appendix

Multimedia Appendix 1 Development and evaluation of a convolutional neural network model for predicting work mode based on human-smartphone interaction data.

## Abbreviations

acti-count: activity count
AUC: area under the curve
GMA: general mental activity
PA: physical activity
WMA: working mental activity

## Data Availability

The data sets generated or analyzed during this study are not publicly available due to restrictions imposed by informed consent from participants in this study, which has been approved by the institutional review boards of the Chang Gung Memorial Hospital. However, they are available from the institutional review board of the Chang Gung Memorial Hospital for researchers who meet the criteria for access to confidential data.
