# Supplementary material for: Developing Methods for Assessing Mental Activity Using Human-Smartphone Interactions: Comparative Analysis of Activity Levels and Phase Patterns in General Mental Activities, Working Mental Activities, and Physical Activities
Source: J Med Internet Res. 2024 Jun 17;26:e56144. doi: 10.2196/56144 (PMC11217705; doi:10.2196/56144)
Supplement: Multimedia Appendix 1 [file jmir_v26i1e56144_app1.docx]

**Supplement. Development and Evaluation of a CNN Model for Predicting Work Mode Based on Human-Smartphone Interaction Data**

1. Data Format Transformation

After passing through the initial XGBoost model, user data outputted the probability of being in a work state at a given time point. We set the threshold at 0.005 and calculated the length of time where the probability difference before and after each point was less than this threshold, using these values as grayscale intensities for the corresponding times. This transformation mapped each time point to a probability value from the XGBoost model and an associated grayscale intensity. Data were then transformed into 30x30 grayscale images at 30-minute intervals, considering data 15 minutes before and after the target time point. Horizontally, the axis represented time, while vertically, the height corresponded to 30 times the XGBoost probability values (scaling [0, 1] to image height [0, 30]), with color reflecting the grayscale intensity.

1. CNN Model Architecture

In this study, we adopted ResNet-34 as the backbone for our convolutional neural network, initiating with pretrained weights. Following the backbone, we appended a fully-connected layer of size two to serve as the model's output. The CNN's backbone primarily functioned to extract image features, which were then utilized to predict the probabilities associated with work activities.

1. Model Fine-tuning

The fine-tuning process of the model consisted of two phases. In the first phase, we froze the backbone weights, focusing the training solely on the newly added fully-connected layer. In the second phase, we gradually trained the final layers of the backbone network, tailoring the extracted features more closely to the characteristics of the individual user.
